# Potential Applications of Rare Earth Metal Nanoparticles in Biomedicine

**DOI:** 10.3390/ph18020154

**Published:** 2025-01-24

**Authors:** Svetlana A. Titova, Maria P. Kruglova, Victor A. Stupin, Natalia E. Manturova, Ekaterina V. Silina

**Affiliations:** 1 I.M. Sechenov First Moscow State Medical University (Sechenov University), 119991 Moscow, Russia; honey.liebe@mail.ru (S.A.T.); silinaekaterina@mail.ru (E.V.S.); 2 Pirogov Russian National Research Medical University, 117997 Moscow, Russia; stvictor@bk.ru (V.A.S.); manturovanatali@yandex.ru (N.E.M.)

**Keywords:** nanoparticles, rare earth metals, lanthanides, rare earth oxides, contrast agents, antitumor effect, antibacterial effect, antioxidant effect, regenerative effect

## Abstract

In recent years, the world scientific community has shown increasing interest in rare earth metals in general and their nanoparticles in particular. Medicine and pharmaceuticals are no exception in this matter. In this review, we have considered the main opportunities and potential applications of rare earth metal (gadolinium, europium, ytterbium, holmium, lutetium, dysprosium, erbium, terbium, thulium, scandium, yttrium, lanthanum, europium, neodymium, promethium, samarium, praseodymium, cerium) nanoparticles in biomedicine, with data ranging from single reports of effects found in vitro to numerous independent in vivo studies, as well as a number of challenges to their potential for wider application. The main areas of application of rare earth metals, including in the future, are diagnosis and treatment of malignant neoplasms, therapy of infections, as well as the use of antioxidant and regenerative properties of a number of nanoparticles. These applications are determined both by the properties of rare earth metal nanoparticles themselves and the need to search for new approaches to solve a number of urgent biomedical and public health problems. Oxide forms of lanthanides are most often used in biomedicine due to their greatest biocompatibility and nanoscale size, providing penetration through biological membranes. However, the existing contradictory or insufficient data on acute and chronic toxicity of lanthanides still make their widespread use difficult. There are various modification methods (addition of excipients, creation of nanocomposites, and changing the morphology of particles) that can reduce these effects. At the same time, despite the use of some representatives of lanthanides in clinical practice, further studies to establish the full range of pharmacological and toxic effects, as well as the search for approaches to modify nanoparticles remain relevant.

## 1. Introduction

At present, there are a number of primary and urgent problems in modern healthcare that require new solutions and approaches. Such problems include the issues of diagnostics of malignant neoplasms and their therapy [1,2], the growth of antibiotic resistance to modern antibacterial drugs [3], as well as the issues of regeneration in skin and soft tissue damage, especially in cases of decreased immune resistance as in diabetes mellitus, tissue ischemia, and many others [4,5]. 

One of the promising directions for solving a number of these problems is the use of nanoparticles of rare earth metals. The widely developing field of nanotechnology, which is already widely used in various spheres [6,7,8,9,10], can be considered as an alternative platform for the development of new therapeutic agents for solving a number of urgent biomedical tasks [11,12]. 

Rare earth metals include metals with atomic masses from 57 to 71 atomic mass units (a.m.u.), as well as 21 and 39 a.m.u. Representatives of rare earth metals are characterized by variable oxidation degrees, which determines their redox properties [13]. The study of the possibility of their application in medicine is a relatively new direction, and it is limited by a number of factors such as the complexity of the synthesis process with the preservation of nanoscale, requiring special conditions and specific equipment [14,15], and most importantly, contradictory data on acute and chronic toxicity of metal nanoparticles [16]. But despite this, some rare earth metals have already gone beyond research interest and have been introduced into clinical practice [17]. For example, gadolinium is currently used as a contrast agent in diagnostic manipulations [18], europium is a promising candidate for the production of drugs for photodynamic therapy of glioma, breast cancer, and cervical cancer [19,20].

The most studied to date are cerium dioxide nanoparticles (nanoceria). According to the international database ScienceDirect, the number of publications on the query “cerium oxide nanoparticles”, devoted to their biological effect, is 3069. More than 1800 of them are represented by original research. Other representatives of this chemical group are much less discussed in the medical and pharmaceutical literature. Thus, the number of publications on biomedical applications of lanthanides in the ScienceDirect database is represented by the following number of articles on similar requests: lanthanum is devoted to 106 publications, praseodymium to 25, neodymium to 48, gadolinium to 200, europium to 53, ytterbium to 18, holmium, lutetium, erbium, terbium, prometium, dysprosium, samarium to 11, 6, 18, 23, 0, 22, and 48 articles, respectively. The biological effects of yttrium, scandium, and thulium oxide nanoparticles are even more poorly discussed in the literature.

However, the question of safety and at the same time preserving or even enhancing the effectiveness of lanthanide nanoparticles, and therefore the possibility of their wider application, is still acute, and it depends not only on their own properties, but also on the possibility of using a number of modifications to ensure the realization of the necessary effects. First of all, it is the chemical composition. A general group feature of rare earth metal nanoparticles is the possibility of their use in the form of oxides characterized by a lower risk of unwanted reactions on the part of living organisms. Compared to other forms of nanoparticles, oxides have on average an order of magnitude smaller size, which promotes better permeability through cellular barriers [21,22]. Other modification methods include changing the degree of oxidation [23], using excipients (particularly polymers) [24], modifying morphology [25], preparing nanocomposites [26] and bioconjugation [27]. All of these may allow their use for biomedical purposes more widely in the future with a relevant level of safety.

**Figure 1:**
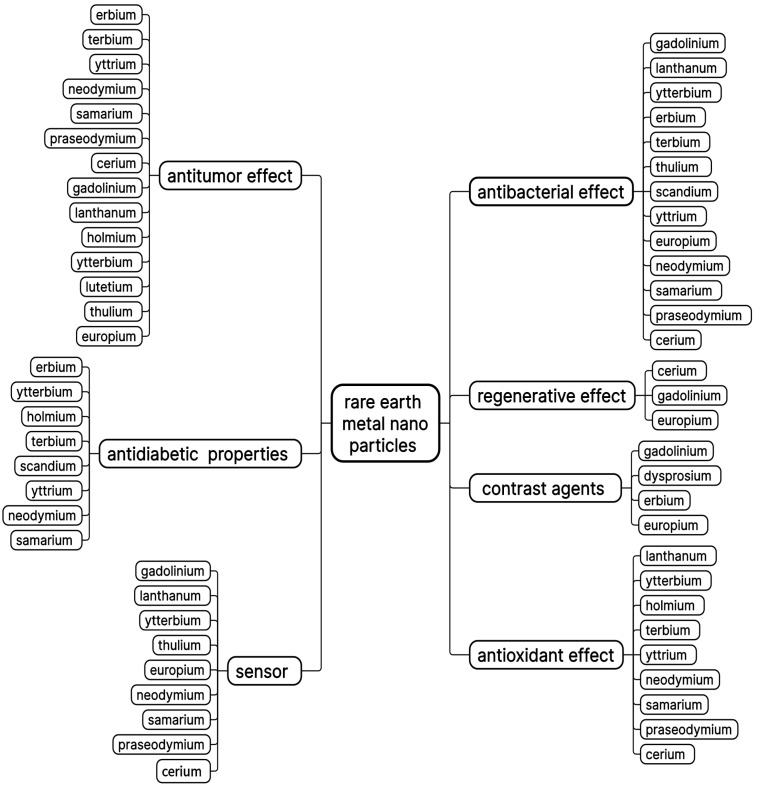
Rare earth metal nanoparticles as agents for biomedical applications.

Thus, the purpose of this review is to summarize the existing topical applications of rare earth metal oxide nanoparticles in biomedicine, characterize each of them in terms of biological activity, focusing on little-known representatives, as well as to outline possible prospects and limitations for this group of elements and existing methods of their modification. When writing this review, we analyzed publications of medical and pharmaceutical topics in the international databases ScienceDirect and PubMed. A brief characterization of rare earth metal nanoparticles in accordance with the objectives of our work is shown in Figure 1.

## 2. Nanoparticles of the Yttrium Subgroup

The yttrium subgroup includes such rare earth metals as gadolinium, ytterbium, holmium, lutetium, dysprosium, erbium, terbium, yttrium, scandium, and thulium.

### 2.1. Gadolinium Nanoparticles

At present, gadolinium nanoparticles of different compositions have been developed: bisphosphonate [28], phosphate [29], gadolinium ascorbate [30], multicomponent nanocomposites [31], and gadolinium oxide nanoparticles, which are the most studied [32,33]. In current clinical practice, gadolinium drugs are represented by contrast agents such as Magnevist^®^, Omniscan^®^, and Gadovist^®^ [34,35,36]. Despite high imaging efficacy, the use of registered gadolinium preparations is associated with a significant risk of severe adverse reactions such as nephrogenic systemic fibrosis [37,38]. The mechanism of development of this phenomenon is associated with the ability of gadolinium compounds to dissociate and further accumulate in body tissues [39]. The use of a non-ionized form of gadolinium, particularly an oxide form, has been proposed as a possible solution to this problem.

Gadolinium oxide is a stable gadolinium (Gd) derivative that exhibits structure polymorphism depending on the method of synthesis and the use of doping materials [40]. In an in vivo comparative study with mice by Dai Y. et al. [41], PEGylated gadolinium oxide nanoparticles had less hepato- and nephrotoxic effects compared to Magnevist^®^. At the same time, the efficiency of tumor tissue visualization exceeded that of the reference drug. However, it should be noted that it cannot be clearly established whether the observed changes in the efficacy and safety profile are related to the nanoscale of gadolinium or to its modification by the excipient [41,42]. A similar result was achieved when compared with Omniscan^®^, which was also confirmed in in vitro and in vivo experiments [43]. Other possible pharmaceutical compositions aimed at reducing the risks of gadolinium-containing agents also include biocompatible polymers in the composition—such as polycyclodextrin [44], dextran [45,46], polyacrylic acid [47], or polymethylvinyl ether of maleic acid [48], for example. In addition to numerous in vitro studies, the safety of formulations containing biocompatible polymers and gadolinium oxide has been confirmed in an experiment using nephrectomized rats. Hanieh Ashouri et al. concluded that due to such modifications of nanoparticles, minimal accumulation of gadolinium can be achieved even in patients with kidney disease [44].

However, current research is not limited to the development of safer forms of gadolinium for medical use. For example, the already described combination of gadolinium oxide and polyacrylic acid nanoparticles in complex with arginylglycylaspartic acid increase targeting of tumor cells by increasing permeability through blood vessels and increased accumulation in cells, which was confirmed by comparing accumulation in normal NCTC1469 liver cells and the U87M glioblastoma cell line in a mouse model [49]. Another illustration of the promise of gadolinium nanoparticles as an antitumor agent is the study by Shailja Kumar et al., in which in addition to surface modification by PEGylation, the nanoparticles were loaded with doxorubicin. Efficacy was demonstrated in in vitro experiments using tumor cell cultures of the A-549 alveolar carcinoma, PANC-1 pancreatic carcinoma, and U-87 glioblastoma cell lines [50]. Comparable results were presented with the improved combination of gadolinium oxide and doxorubicin. This composition was supplemented with β-cyclodextrin and folic acid, giving the properties of a system with pH-sensitive release of the active ingredient, extending the spectrum of potential applications to solid tumors [51].

Another area of research into the possibility of using gadolinium oxide in oncology was the study of the therapeutic properties of this nanoparticle [52]. A number of publications have described its radiosensitizing properties [53,54]. For example, the 2019 work demonstrated activity against B16F10 melanoma cells [55]. A possible mechanism explaining this property is the established induction of oxidative stress causing DNA damage. According to the researchers’ conclusion, these damages exceeded the fragmentation caused by isolated irradiation by 52% [56]. An important feature is the retention of this effect when attempting to target glioblastoma cells not only in the GL261 cell line [57], but also in vivo, although with the need for additional functionalization [58]. Its ability to affect the reduction in PTX3 protein expression was also established, which indirectly indicates the possibility of slowing down the metastatic process [59]. However, it should be noted that the described properties are dose- and time-dependent, which, together with the ability to pass through the blood–brain barrier, may complicate the prediction of the intensity of apoptosis and necrotic processes when trying to apply this information in clinical practice [58,60,61]. This observation brings us back to the question of the need to balance risk and benefit in the use of metallic nanoparticles.

It should be noted that the prospects for the use of gadolinium oxide nanoparticles as a drug go beyond the diagnosis and therapy of malignant neoplasms [54,62,63,64,65,66]. In modern literature it is reported that gadolinium has antimicrobial properties. According to the results of studies, it exhibits antibacterial activity against *Escherichia coli*, *Staphylococcus aureus*, *S. Typhimurium*, and antifungal activity against *Candida albicans*, *C. glabrata*, and *Fusarium oxysporum* [67,68,69]. Also, according to 2023 studies, gadolinium oxide can be used as an immunosensor to detect Vibrio cholerae toxin [70,71].

One of the promising and relevant areas of application of gadolinium oxide nanoparticles is its possible application in the field of regenerative medicine. In the studies of Vijayan V. et al., it was found that gadolinium oxide in the hydrogel of collagen and dextran shows anti-inflammatory and regenerative properties in corneal explant and inhibits neovascularization in endothelial cells of umbilical vein EA.hy926 [72]. In addition, bisphosphonate-functionalized gadolinium nanoparticles were found to be suitable as bone tissue engineering agents. At concentrations up to 10 × 10^−6^ m, the nanoparticles induced cell proliferation of primary human osteoblast cells, promoting bone regeneration. The efficacy was confirmed by in vivo MRI imaging. [73]. Large-scale studies conducted on human cell cultures have established the antioxidant activity of gadolinium oxide nanoparticles, acceleration of the metabolism of fibroblasts BJ TERT (by 27%), and proliferation of keratinocytes HaCaT (by 35%) in a wound surface model [74]. It was also found that when gadolinium was incorporated into a hydrogel containing the biopolymers dextran, collagen, and gelatin, a synergistic regenerative effect ex vivo was achieved [72]. However, the application of gadolinium in the above directions requires further research. A significant feature of gadolinium oxide nanoparticles is the fact that no cytotoxic effects were found in studies using cultures of SK-MEL-28 melanoma cells, adipose-derived mesenchymal stromal cells (ASC) [45], A-549 lung carcinoma cells, U-87 glioblastoma cells, HEK-293 normal cells [46], and the DU145 human prostate cancer cell line [48].

Thus, at the moment, the most studied and widespread application of gadolinium nanoparticles is MRI diagnostics and therapy of malignant neoplasms.

### 2.2. Ytterbium Nanoparticles

Ytterbium is most often used in biomedicine in the form of oxide, which is a compound that has a variable morphology depending on the method of synthesis, as well as the ability to luminescence [75]. A significant number of references to ytterbium oxide nanoparticles in the literature are devoted to their use as a potential antitumor agent [76,77,78]. It should be noted that ytterbium oxide has not been studied in isolation and was usually an element of complex stimulus-sensitive compositions. Thus, in the experiment of Peng H. et al., performed on MCF-7 breast adenocarcinoma cell cultures, ytterbium was part of a multicomponent shell that also included β-cyclodextrin, zinc oxide, and erbium deposited on an iron oxide core. According to researchers, this shell provided microwave-sensitive release of the active ingredient accompanied by controlled toxicity [77]. In a study with a different model drug, camptothecin, loaded on ytterbium ferrite nanoparticles coated with a polylactide-co-glycoside functionalized with a β-cyclodextrin-folate conjugate, a pH-dependent release of the active component was detected, which increased significantly in the slightly acidic environment characteristic of the tumor microenvironment. It was found that this composition has the ability for sustained prolonged release targeting folate receptors, but the results were also limited to in vitro experiments [78].

Special attention should be paid to the existing publications that have revealed antimicrobial properties of ytterbium oxide nanoparticles. The greatest contribution in this direction was made by the team of V. Muthulakshmi et al. who carried out a series of large-scale experiments in vitro. According to their results, ytterbium oxide exhibited an antibacterial effect against *E. coli* and *S. aureus*. The level of activity depended on the presence of a nanoparticle coating. Thus, when 1-butyl-3-methylimidazolium hexafluorophosphate was added, the result was superior to the activity of amikacin [79,80]. In other works, developing this direction, the possibility of using ytterbium oxide with chitosan as a dressing material was investigated. According to the results, the presence of antibacterial activity against Gram-positive and Gram-negative bacteria was confirmed, as well as the biocompatibility of this composition [81]. In addition to the study of antimicrobial action, V. Muthulakshmi et al. identified the potential use of ytterbium nanoparticles as anti-inflammatory, antioxidant, and diabetes drugs. Despite the fact that the effect of coated nanoparticles was superior to that of reference drugs (in particular, diclofenac and ascorbic acid), at the moment it is difficult to assess the real potential since the experiments were carried out under in vitro conditions and require further development of these directions [79,80].

In modern scientific publications, ytterbium oxide nanoparticles are considered not only as a potential therapeutic agent, but also as a diagnostic agent for a number of pathological conditions. To date, electrochemical sensors have been developed to determine blood glucose levels [82], dopamine [83], including dopamine and uric acid in the presence of ascorbic acid [84], and methadone [85].

A number of publications provide data on the cytotoxicity and antiproliferative effect of ytterbium. Thus, in the study of Wysokińska E. et al. for mouse embryonic fibroblast cells NIH3T3 and mouse macrophages RAW264.7 it was revealed that a significant loss of viability was observed at nanoparticle concentrations of 26 μg/mL and 1.6 μg/mL, respectively, although it should be noted that coating nanoparticles with polyethylene glycol reduced this effect, although it did not get rid of the inhibitory effect on proliferation [86]. Similar results were obtained in studies conducted on human hepatocellular carcinoma HepG2 cells. Wang C. et al. identified the mitochondrial-mediated pathway as a putative mechanism of apoptosis. The scientists also noticed that the toxicity of ytterbium oxide was more dependent on its surface properties rather than particle size [87]. Data from published in vivo studies report additional unwanted effects of ytterbium oxide. In particular, when administered through the respiratory tract, it induced oxidative stress in the lungs of mice with a histological picture of chronic inflammation [88].

In the course of analyzing the above-mentioned works, we can conclude that the application spectrum of ytterbium in biomedicine (mainly antitumor and antimicrobial) is rather narrow, which is determined by the data on its cytotoxic properties.

### 2.3. Holmium Nanoparticles

Holmium nanoparticles are represented by phosphate [89], acetylacetonate [90], and holmium oxide [91]. Their biological effects are poorly reported in the literature. The attention of the modern scientific community is more focused on the selection and standardization of optimal synthesis methods [92].

At present, it is known that chemically unmodified holmium oxide particles are capable of cumulation in the liver and spleen, as well as induction of immunologic reactions in vivo [93]. Also, like other lanthanides, it can be a potential candidate for the development of diagnostic imaging agents [94]. There are also references to its pharmacological action. In an in vivo study by Luo Y. et al. it was found that holmium oxide nanoparticles promoted angiogenesis by regulating the EphrinB2/VAV2/CDC42 signaling pathway, which also led to improved regeneration on the model of bone defects in rats [95]. On the other hand, Ahmed Mohamed H. E. et. al. performed experiments on cultures of MCF-7 ductal adenocarcinoma breast adenocarcinoma cells, 3T3 mouse embryonic fibroblasts, and in ovo experiments to determine the antioxidant and antidiabetic potential and revealed antiangiogenic properties [96]. Thus, the role of holmium oxide as a regulator of angiogenesis remains unclear.

One of the current areas of development is the use of the holmium-166 isotope in topical dosage forms, patches and medical devices (nanofiber dressings), for the therapy of squamous cell skin cancer [97,98,99]. Under in vivo conditions, a statistically significant increase in survival rate and reduction in tumor size were achieved [99].

Information on modified holmium oxide nanoparticles is also quite contradictory. In particular, the toxicity of their compounds with various polymers has been investigated. For example, in a study using DU145 prostate cancer cell cultures, the cytotoxicity of nanoparticles coated with polyethylenimine 1200 and polyethylenimine 60,000 was evaluated. After 48 hours, the signs of cytotoxicity were minimal. At the same time, free polyethylenimine decreased the percentage of viable cells by 22% [100]. Experiments evaluating PEGylated holmium oxide also demonstrated a favorable toxicity profile for L-929 fibroblasts at nanoparticle concentrations within 16 μg/mL [101].

Thus, the available data are insufficient to draw an unequivocal conclusion, for example, because of the presence of opposing effects regarding angiogenesis. However, potentially possible chemical modifications of holmium may be promising and provide the possibility of its application in biomedicine.

### 2.4. Lutetium Nanoparticles

Lutetium nanoparticles are usually represented either by multicomponent nanocomposites [102,103,104] or lutetium oxide. An interesting feature of publications devoted to the medical use of lutetium is the presence of data on the properties of its isotopes, in particular, lutetium 177 [105,106]. A study by Luna-Gutiérrez M. et al. evaluated its use as a radiopharmaceutical for intra-arterial administration, which provided a 7.5-fold reduction in prostate tumor size relative to the control group [105]. A similar study was carried out with hepatocellular carcinoma HepG2 cells. Additionally, it revealed a preferential accumulation of the isotope in the liver and spleen, which is in full agreement with the data obtained for other rare earth metals [106].

Regarding the cytotoxicity of lutetium, there are data on its effects on endothelial cells. The cytotoxicity of lutetium oxide to human HECV endothelial cells was investigated at concentrations ranging from 0.1 µg/mL to 10 µg/mL. The absence of toxic effects was found only with the lowest tested concentrations. However, it should be noted that the nanoparticles were doped with samarium; therefore, its influence on the result cannot be excluded because of the lack of control samples without samarium in the cytotoxicity study [107]. Despite this, during the study of lutetium nanoparticles in vivo, encouraging results were obtained. The data obtained by Liu Z. et. al. demonstrated not only the suitability of lutetium for use as a CT imaging agent but also the relatively rapid and complete elimination of lanthanide from the mouse body within one month [108].

Thus, information on biomedical applications of lutetium oxide nanoparticles is limited to isolated reports on its toxicity, toxicokinetics, and prospects for the development of its isotope.

### 2.5. Dysprosium Nanoparticles

Dysprosium nanoparticles include phosphate [109] and vanadate [110] in addition to oxide, but biological effects have been studied specifically for the oxide form. Dysprosium nanoparticles have been described as a potential candidate for MRI imaging drugs [109,111,112]. Through carbon coating, Yue H. et al. were able to achieve ultra-small nanoparticles with an average diameter of 3.0 nm and a hydrodynamic radius of 22.4 nm. The coated nanoparticles showed significantly less cytotoxicity than uncoated nanoparticles in an experiment on DU145 human prostate cancer cells and NCTC1469 mouse hepatocyte cells. In the next stage of the experiment, which was carried out in vivo, it was found that dysprosium oxide is an effective tool for visualization, and due to the specifics of its excretion, it can be used for diagnostic manipulation of various organs and tissues [112]. A similar result, including cytotoxicity, was achieved in the synthesis of dysprosium nanoparticles and nanorods conjugated with glucuronic acid. An interesting observation was the fact that nanorods, due to their larger size than nanoparticles, showed a longer period of accumulation in the liver of the experimental mouse, which led to a lethal outcome [113]. Thus, we can make an assumption that nanoparticles, rather than other morphological variants of nano-objects, should be considered as the priority area of research on rare earth metal nanoparticles. When the compositions were further complicated, namely, mixed gadolinium-dysprosium nanoparticles coated with hyaluronic acid, minimal cytotoxicity was maintained and the mixed nanoparticle showed better contrast than isolated gadolinium and dysprosium nanoparticles [114]. Another example of successful development of mixed nanoparticles could be iron oxide-dysprosium oxide particles. The obtained nanoparticles had a diameter of 4 nm and showed satisfactory safety in vitro. In an in vivo study, it was found that the combination of iron oxide and dysprosium oxide, as a diagnostic agent, can reliably differentiate early and moderate stages of liver fibrosis [115].

The literature also contains some reports on the possibility of using dysprosium oxide in other areas of medicine. For example, according to modern studies, it is possible to use dysprosium as an electrochemical detector for standardization and detection of substandard drugs in pharmaceutical activities [116,117].

Thus, dysprosium oxide nanoparticles can be considered as a tool for MRI diagnostics. However, when working with animals and in clinical practice, the need for the addition of excipients that could reduce cytotoxicity cannot be excluded.

### 2.6. Erbium Nanoparticles

As in the case of other lanthanides, data on possible applications of erbium are presented by the application of its oxides. Erbium oxide nanoparticles are a compound averaging 5 to 30 nm and are capable of luminescence [118]. Like other rare earth metals, erbium has been studied to evaluate the possibility of use as an imaging agent in MRI diagnostics. In an in vivo experiment by Tse J. J. et al. it was found that erbium oxide is a suitable agent for visualization of vessels less than 10 μm in diameter, making it one of the most promising candidates for further research in this direction [119].

It should be noted that this nanoparticle also has a fairly wide range of pharmacological activity. For example, several studies have demonstrated its antibacterial effect against *Staphylococcus aureus*, *Escherichia coli*, *Desmodesmus subspicatus*, and *Pseudomonas aeruginosa* with no effect against *Enterococcus faecalis* [120,121]. An interesting observation was the fact that antibacterial activity can be enhanced by additional ultraviolet irradiation [121]. There are also references to the hypoglycemic properties of erbium oxide. They were studied in vitro using α-glucosidase and α-amylase, as well as in vivo, with no signs of acute toxicity in animals [122].

Data about the presence of antitumor effects were also published, but the experiments were conducted only under in vitro conditions [120,123]. In the study by Safwat G. et al. the mechanism of action of erbium oxide nanoparticles on Hep-G2 hepatocellular carcinoma cells was described. They were found to inhibit cell proliferation and induce oxidative stress, resulting in DNA damage leading to apoptosis [123]. Interestingly, when working with the same cell culture, but with additional carbon coating of the nanoparticle, cytotoxicity was minimal [124]. However, it should be noted that according to Mohamed H. R. H. et al., using data obtained by working with HSF fibroblast cell cultures, the cytotoxicity of nanoparticles containing erbium can be characterized as dose-dependent and occurring with prolonged exposure, which requires additional chronic toxicity studies [125].

Antibacterial, antitumor, and hypoglycemic properties have been reported for erbium oxide nanoparticles, but evaluation of the possibility of using erbium in the therapy and diagnosis of diseases requires additional research.

### 2.7. Terbium Nanoparticles

Terbium nanoparticles are nano-objects with the property of fluorescence [126,127,128,129]. The largest-scale experiments devoted to the evaluation of pharmacological effects were conducted by P. Senthil Pandi et al. in 2024. The researchers evaluated anti-inflammatory, antidiabetic, antitumor, and antioxidant activity. According to their findings, terbium nanoparticles demonstrated in vitro efficacy comparable to diclofenac sodium, acarbose, and ascorbic acid [130]. The literature also provides information on the antibacterial activity of terbium oxide against *Escherichia coli* and *Staphylococcus aureus*, as well as regenerative properties in vivo [131].

Most studies on the biological effects of terbium oxide nanoparticles address the issues of its potential antitumor activity. For example, in the experiments of Sana Iram et al. using osteosarcoma cell cultures MG-63 and Saos-2, a significant cytotoxicity was established, which was assumed to be caused by the generation of reactive oxygen species, but no such effect was observed for normal osteoblasts obtained ex vivo [132]. The concentration dependence of toxicity was investigated by Eman I. Khalaf et al. The scientists found that for MCF-7 breast cancer cells and HepG2 hepatocellular carcinoma cells, a significant decrease in viability was observed only at a concentration of 100 µg/mL, which is a sufficiently high dose for nanoparticles [133]. Terbium has also been described as an effective agent of photodynamic therapy, including the results of in vivo experiments [134,135]. In a comparative study of nanoparticles doped with gadolinium and terbium, the latter demonstrated a 35% more pronounced cytostatic effect on U-251 MG glioblastoma cells [136].

Thus, information on the pharmacological effects of terbium oxide is currently insufficient. Given that most of the experiments mentioned in this section have been conducted in the last few years, we can hope that the study of the properties of these nanoparticles by the international scientific community will continue.

### 2.8. Thulium Nanoparticles

Thulium nanoparticles are represented by oxide [137] and hexacyanoferrate II [138]. In modern clinical practice, thulium is used mainly as a fiber laser in urology for lithotripsy and enucleation of the prostate gland [139,140,141,142]. Actual developments of thulium nanoparticles are devoted to their study as a visualization agent for malignant neoplasms, including microscopic ones, due to their up-conversion properties [143,144]. High fluorescent activity was demonstrated by Yang Yiwei et al. in in vivo experiments [145]. Also, thulium oxide nanoparticles, due to their specific uptake by tumor tissue, confirmed in work with models of breast tumors 4175 and MCF-7 and ovarian cancer SKOV3 [146], can be used as a radiosensitizer [137]. It should be mentioned that thulium-containing nanoparticles have selective toxicity against malignant tumor cells, such as those overexpressing HER2 [147], but have no therapeutic effect against GL261 and U-251 MG glioma cells [148]. A possible mechanism for the development of therapeutic action is the ability of thulium oxide to generate reactive oxygen species induced by near-infrared radiation [149,150]. A similar effect underlies the antibacterial properties of thulium nanoparticles demonstrated by Zhou Kun et al. against MRSA [151].

In addition to oncological applications, thulium nanoparticles have been investigated as a sensor. In particular, the possibility of their application as a sensor of glucose [138], diethylsilbestrol, and bisphenol A [152] was described.

It should be noted that in most cases researchers use thulium as a doping material for other nanoparticles rather than as an independent therapeutic or diagnostic agent [143,153,154,155,156].

Thus, despite the lack of confirmed cytotoxic effect [155] and immunogenicity [157], an objective assessment of the possibility of using thulium nanoparticles for medical applications requires additional studies of its activity and kinetics.

### 2.9. Scandium and Yttrium Nanoparticles

In addition to the 15 lanthanides, the group of rare earth metals includes scandium and yttrium. Scandium oxide nanoparticles are currently poorly understood as a means for medical applications. As potential applications of scandium, researchers have proposed nanocomposites with antibacterial properties (against *Escherichia coli*) [158], as well as suitable for noninvasive determination of glucose levels in exhaled air for monitoring diabetes control [159].

The current literature reports that scandium oxide has satisfactory biocompatibility without adverse effects on cell proliferation, which was established when working with the HOS TE 85 osteoblast-like culture line [160]. Assessment of the distribution of scandium in the body, carried out in in vivo experiments, showed that the greatest accumulation of this metal occurs in the liver and lungs, while the cumulation of nanoparticles in the lungs is dose-dependent and exceeds that of soluble forms of scandium [161].

Yttrium has already found application in real clinical practice in the form of the isotope yttrium 90 and is used for selective radiation therapy of malignant liver neoplasms [162], as well as brachytherapy of nonmelanoma skin cancer in combination with strontium 90 [163]. Further development of the direction led to the development of patches loaded with yttrium 90 microspheres, but the assessment of the possibility of application requires additional in vivo studies [164]. It is worth noting that the stable isotope of yttrium can also be applied in this direction, demonstrating therapeutic potential as a means of photodynamic therapy [165].

A significant number of studies conducted on laboratory animals report pronounced antioxidant properties of yttrium oxide nanoparticles [166,167,168,169]. According to the results of in vivo experiments, it can be concluded that the antioxidant activity of nano yttrium can have a therapeutic effect on a number of pancreatic pathologies. For example, Khurana A. et al. found a reduction in oxidative-nitrosative stress in models of both acute and chronic pancreatitis [170,171]. The pursuit of anti-diabetic properties has also led to positive findings [172]. For example, an increase in viability, ATP, and insulin production was observed in the pancreas of rats pretreated with hydrogen peroxide [173,174]. It is noteworthy that the favorable effect was also observed upon administration of diazinon, an organophosphorus insecticide, the main target of which in warm-blooded organisms is the pancreas. Several independent in vivo experiments revealed that yttrium oxide nanoparticles normalize the ATP/ADP ratio and the activity of caspases 3 and 9 and reduce the manifestations of oxidative stress caused by diazinon [175,176].

The potential application of antioxidant properties of yttrium oxide nanoparticles is not limited by the therapy of pancreatic diseases. Almost a comparable number of studies are devoted to the neuroprotective effects of nano yttrium. Initially demonstrated on cultures of hippocampal HT22 and pheochromocytoma PC12 nerve cells, an antioxidant effect [177,178] was confirmed in vivo. In the work of Baghaee P. et al., a weakening of the manifestation of cognitive deficits in the model of Alzheimer’s disease was noted, presumably due to the reduction in mitochondrial dysfunction and neuroinflammation [179]. Neuroprotective effects were also achieved in models of lead [180] and silver oxide [181,182] poisoning, as revealed by assessing caspase 3 expression, Bax/Bcl-2 proteins, ADP/ATP ratio [180], and levels of oxidative biomarkers (such as superoxide dismutase levels, glutathione, etc.), as well as neurotransmitters (acetylcholinesterase, dopamine, and serotonin) in the brains of mice [181] and people with behavioral disorders [182].

Other prospects for the use of yttrium oxide nanoparticles are currently less studied. The literature describes the possibility of using yttrium nanoparticles in retinal degeneration in vivo [183], and they also demonstrate a selective and intense cytotoxic effect against MDA-MB-231 triple-negative breast cancer cells, which reports antitumor potential [184], as well as a means for visualization of malignant neoplasms [185].

It should be noted that yttrium oxide nanoparticles are characterized by high biocompatibility, even at a dosage of 200 µg/ml/day [186,187]. Some studies describe manifestations of cytotoxicity against HEK293 embryonic kidney cells [188] and neurotoxicity by astrocyte cuproptosis in vivo [189]. At the same time, most researchers are inclined to the fact that the use of yttrium nanoparticles is associated with a low risk of unwanted effects.

Thus, the most studied effects for scandium nanoparticles are antibacterial, and the most studied effects for nanoyttrium nanoparticles are antioxidant.

## 3. Nanoparticles of Metal Oxides of the Cerium Subgroup

The cerium subgroup includes lanthanum, europium, neodymium, promethium, samarium, praseodymium, and cerium.

### 3.1. Lanthanum Nanoparticles

Lanthanum nanoparticles are a heterogeneous group of compounds, including hydroxides [190,191] and borides [192,193], possessing variability of morphology [194] and exhibiting scintillation [195]. This property prompted scientists to investigate the possibility of using lanthanum nanoparticles as antitumor agents. The work of Lu V. M. et al. presented data on the ability of lanthanum oxide nanoparticles to cross the blood–brain barrier during intravenous administration, as well as the manifestation of cytotoxicity against glioblastoma cells by generating reactive oxygen species, which was demonstrated in an in vivo experiment [196]. Similar results were demonstrated for PEGylated lanthanum nanoplatforms. In this study, also performed on an in vivo model of glioblastoma, the sensitivity of the composition to the pH of the tumor microenvironment and a higher efficiency of the damaging effect due to an increase in the specific surface area of the nanoparticle were revealed [197]. On the other hand, lanthanum nanoparticles can be considered not only as an independent antitumor agent, but also as a delivery system. In particular, the use of systems containing lanthanum and various radium isotopes for targeting micro metastases has been proposed [198].

Given the risk of systemic reactions, an alternative application of lanthanum nanoparticles in medicine may be its use as a local agent. Published studies report its antibacterial (*S. aureus* and *E. Coli*) and antioxidant properties that have been demonstrated in vitro, as well as a relatively small antifungal effect [199,200].

In addition to the therapeutic potential, lanthanum hydroxide nanoparticles coated with cysteine have been investigated as an electrochemical sensor for the diagnosis of oral carcinoma by detecting the marker Cyfra-21-1 [201]. Perhaps priority should be given to developing the potential of lanthanum nanoparticles as a diagnostic agent, as the mechanism of the identified antitumor action may be an issue in future attempts to apply lanthanum nanoparticles in clinical practice. The reason for this may be the fact that similar negative effects were detected both in HuH-7 liver tumor cells and in normal CHANG cell lines, however, with more severity in the former case [202,203]. Toxic effects of lanthanum have also been demonstrated in in vivo studies. When lanthanum oxide was injected into mice, a significant increase in the levels of superoxide dismutase, glutathione peroxidase, and caspase-3—markers of oxidative stress—was observed. The damaging effect was also proved by histological examination of the liver [204]. When administered orally, cumulation of lanthanum in bone tissue at a depth of up to 5 μm was noted [205]. However, in the study by Vijayan V. et al., also conducted in vivo, favorable effects were presented, indicating the regenerative effect of lanthanum-containing nanocomposites on bone tissue [206].

It should be noted that there are published data on the toxic effects of lanthanum in studies involving people whose activities were related to work with rare earth metals, with longer contact (5 years or more) leading to their cumulation in the body [207,208]. A special cause for concern is potential neurotoxicity. In vivo experiments have shown that lanthanum oxide nanoparticles can cause behavioral disorders, damage to hippocampal neurons, and a decrease in plasma levels of norepinephrine and acetylcholine [209,210]. As a possible way to reduce this toxic effect, Lu Yuan et al. proposed the use of an antioxidant from the group of carotenoids, which, according to the authors, had a neuroprotective effect due to the induction of the PI3K/AKT signaling pathway [210].

Despite the available data on cytotoxicity, including neurotoxicity, lanthanum can be considered as a promising antitumor agent, such as in the case of glioblastoma, which remains the most common malignant neoplasm of the brain with an extremely poor prognosis and limited possibilities of existing therapy [211]. On this point, we believe that the ratio of potential benefits to risks is a sufficient incentive to continue development in this area.

### 3.2. Europium Nanoparticles

Europium nanoparticles are a compound with luminescent properties and regulated photocatalytic activity [212]. Nanoeuropium has been investigated in many areas of biomedicine. As for other lanthanides, studies of europium as a tool for MRI imaging of tumors have been described in the literature [213].

Further development of the field led to experiments devoted to evaluating the possibility of using these nanoparticles as a radiopharmaceutical to visualize tumor tissue during its surgical removal. In a study by Zhenhua Hu et al. using a tumor model in laboratory mice, it was described that the use of europium allows the detection of neoplasms smaller than 1 mm, as well as residual carcinoma tissue without histological signs of toxicity [214]. Less encouraging data are presented in the work of Min Li et al. It confirms the information about the preferential accumulation of lanthanide nanoparticles in the liver and spleen during injection, but at the same time, there was an observed change in the population of immune cells in vivo, which, accordingly, increased the risk of unwanted reactions [215]. One of the possible directions for solving the problem of potential systemic toxicity is precisely the local use of europium nanocomposites as a means for photodynamic therapy of skin cancer when combined with photosensitizers, such as methylene blue [216,217,218]. Interestingly, the europium content in the nanocomposite and the radiation intensity were not directly related, and in the concentration range from 1 to 50%, the highest value was observed for 10% [219].

A safer variant may be the use of europium oxide nanoparticles for topical application. For example, as an antibacterial agent. The antimicrobial activity of europium oxide was found against Gram-positive and Gram-negative bacteria *E. coli*, *S. aureus*, and *S. typhimurium* [220]. At the same time, europium in nanocomposites was also found to exhibit antioxidant and regenerative properties, making it a potential candidate for the development of topical wound healing agents [221,222,223]. 

Another possible application of europium nanoparticles in medicine is as an electrochemical sensor. In a study by Tuğçe Teker et al. a carbon platform with the addition of dysprosium and europium oxides showed high sensitivity and selectivity in the determination of papaverine not only in pure form but also in biological fluids, which possibly makes it suitable for use in pharmaceutical chemistry and toxicology [224].

Based on the above, the application of europium oxide nanoparticles as an agent for visualization of malignant neoplasms is the most studied one.

### 3.3. Neodymium Nanoparticles

Based on the available data, neodymium is also used predominantly in the form of oxides. Neodymium oxide is a nano compound with photocatalytic properties and, depending on the method of synthesis, can exist as nanoparticles prone to aggregation or nanosheet formation [225]. The structure, optical properties, and morphology are influenced by the type of precursor [226] as well as the use of polyelectrolytes for stabilization [227]. Due to the sufficiently deep study of this topic, it became possible to use neodymium nanoparticles to produce hydrogel contact lenses, which in combination with methacrylic acid demonstrated high strength and optimal transparency [228]. Another promising example of the application of neodymium oxide as a medical device is an electrochemical sensor for the determination of adrenaline and tyrosine in drugs and biological samples. According to Biswas S. et al., this sensor is characterized by high specificity, selectivity, and repeatability of measurement results, and as a consequence, it can be used in clinical practice [229].

The therapeutic effect of neodymium, according to the current literature, although poorly studied, is characterized by a fairly wide spectrum. Muthulakshmi V. et al. in an in vitro study from 2022 report the presence of neodymium oxide antibacterial (against *E. coli* and *S. aureus*), antidiabetic, anti-inflammatory (determined in a test with bovine serum albumin), antioxidant, and antitumor properties (when working with MCF-7 breast adenocarcinoma cells) [230]. Comparable results were reported from experiments by M. Sundrarajan V. et al. conducted under similar conditions, indicating a satisfactory comparability of data [231]. Despite the achievement of all previously described effects, in vivo studies are required to confirm the acceptability of neodymium as an active pharmaceutical substance. A possible barrier to further development of drugs based on neodymium oxide is information about its toxicity. It was found that inhalation administration of neodymium oxide nanoparticles in bronchial epithelium produces an inflammatory reaction accompanied by an increase in the expression of interleukins 6 and 8, as well as an increase in the level of lactate dehydrogenase [232]. Also, the results of several studies have reported the ability of neodymium oxide to have a damaging effect on the DNA of the bronchial epithelium. It is noteworthy that the conclusion about the adverse effects of neodymium was based not only on the findings obtained when working with cultures of human bronchial epithelial cells 16HBE or laboratory animals, but also on the basis of examinations of people (a sample of 60–69 people) working with these nanoparticles [233,234].

Thus, neodymium oxide nanoparticles can be considered as a potentially effective antibacterial, antioxidant, antitumor, and anti-inflammatory agent, but more research is required to make a definitive conclusion on their acceptability for medical use.

### 3.4. Promethium Oxide

Promethium is characterized by high radioactivity, which limits its application [235]. Data on the biological properties of its oxide nanoparticles are limited by information about its ability to cause alveolar proteinosis when administered intratracheally to laboratory animals, which, however, is not its specificity, since a similar reaction is also observed for europium, samarium, dysprosium, and terbium oxides [236]. Accordingly, the use of promethium nanoparticles in medical practice is currently not possible.

### 3.5. Samarium Nanoparticles

Samarium nanoparticles are represented by various nanocomposites [237,238,239] as well as samarium oxides. The most studied is the effectiveness of samarium oxide nanoparticles as an antibacterial agent. This effect was found against such microorganisms as *Escherichia coli*, *S. aureus*, *Bacillus subtilis* [240,241], as well as *Pseudomonas aeruginosa* [242]. A fungicidal effect was also revealed for a wide range of microscopic fungi such as *Alternaria brassicae*, *Fusarium acuminatum*, *Aspergillus flavus*, *Aspergillus penicillioides*, *Candida albicans*, and many others. It should be noted that the independent antimicrobial activity of samarium oxide is less than the effect of silver nanoparticles, but in the composition of complex nanoparticles, silver and samarium show greater efficacy [241]. It is possible that the role of samarium is not limited to the realization of mechanisms characteristic of rare earth metals [241] but is also associated with its ability to inactivate efflux by affecting the MFS genes encoding transfer proteins [243].

The literature also describes antidiabetic, antitumor, anti-inflammatory, and antioxidant properties of samarium oxide nanoparticles, determined in vitro, and, in general, demonstrating the maintenance of the general trend to determine these properties of rare earth metal nanoparticles in the scientific environment in recent years [240,244]. For example, samarium is currently used in the form of the radiopharmaceutical samarium 153 for the treatment of metastatic lesions [245].

Of greater interest is the study by Navid Mohammadian et al. in which samarium oxide was proposed as a specific and highly sensitive electrochemical sensor for the detection of the gene responsible for 20% of cases of amyotrophic lateral sclerosis; this was also tested in blood plasma, which indicates its potential in diagnosis [246]. At the same time, we cannot fail to mention the cytotoxic properties of samarium oxide nanoparticles, revealed when working with MC3T3-E1 preosteoblast cells. It is currently unknown whether this problem can be completely solved by the creation of complex nanoparticles, as was performed in the experiment of Milli Suchita Kujur et al., or by the use of samarium in ultralow doses [247].

The most studied effect for samarium nanoparticles is the antimicrobial effect, but based on research in recent years, we can assume that the potential range of applications will be expanded in the near future.

### 3.6. Praseodymium Nanoparticles

Praseodymium nanoparticles can be presented not only as oxide but also as hydroxide [248], and they can be modified with other metals or polymers. The antitumor properties of praseodymium nanoparticles are most frequently mentioned in the literature. In a study by Bakht M. K., praseodymium is characterized as a potential dual therapeutic agent in non-small cell lung cancer. It was found that it can be used as a radiopharmaceutical drug that also induces massive tumor cell death [249]. Additionally, loading of nanoparticles with doxorubicin with its controlled release and intensification of the effect due to photothermal transformation of the particle is possible [250]. In the experiment of Varnitha Manikantan et al. conducted on cultures of MCF-7 breast adenocarcinoma cells and Vero kidney epithelium, it was revealed that praseodymium oxide coated with poly-β-cyclodextrin is able to provide selective cytotoxic effects of 5-fluorouracil for tumor cells [251]. In addition, praseodymium can also be used as a diagnostic tool for malignant neoplasms. For example, Zhang Q. C. et al. investigated the possibility of its use as a gas sensor to detect traces of breast cancer marker (acetophenone) in exhaled air [252].

Praseodymium oxide nanoparticles have also been studied in other areas of medicine. For example, activity has been found against a wide range of microorganisms such as *S. aureus*, *E. coli*, *P. aeruginosa*, and *S. typhi* [253]. In the current literature, there are publications reporting evidence of regenerative [254] and antioxidant [255] properties of praseodymium nanoparticles, but they were part of complex compositions that also included collagen [254] and cerium [255], so it is impossible to make a definitive conclusion regarding the effect of a particular component.

Thus, praseodymium oxide nanoparticles can be characterized primarily as a potential antitumor agent as well as a drug for diagnosis. In addition, its antibacterial effects should also be considered as promising.

### 3.7. Cerium Nanoparticles

As a potential agent for medical applications, the modern scientific community primarily considers cerium oxides [256,257,258,259,260,261,262,263]. Due to the antioxidant properties of nanoceria, it has therapeutic potential in a wide range of pathologies. Thus, positive effects have been demonstrated in a number of studies: hepatoprotective effect [264] on models of carbon tetrachloride poisoning, non-alcoholic fatty liver disease of rats [265,266,267] and doxorubicin-induced lipid peroxidation [268], reduction in functional and biochemical manifestations of pulmonary hypertension and cardiomyocyte apoptosis in a model of pulmonary hypertension [269], long-term protection against acute damage to retinal neurons [270] and ischemic hippocampus [271], and radiation-induced lung injury [272].

Antioxidant action is also the basis of regenerative properties of cerium dioxide. Thus, during experiments on human fibroblast cultures, a dose-dependent induction of cell proliferation was established, as well as intensification of metabolism, manifested by an increase in elastin and collagen synthesis without signs of cytotoxicity [273]. Similar results were achieved when working with cultures of mesenchymal stem cells and keratinocytes [274]. Moreover, efficacy has been demonstrated ex vivo on spinal cord tissue subjected to mechanical traumatization [275], for non-sterile wounds in vivo [276], and in numerous experiments reporting intensification of osteogenesis, cell differentiation, and mineralization [277], including in periodontal regeneration [278,279]. Thus, we can consider cerium dioxide nanoparticles as a potential agent for regeneration of sterile and contaminated wound surfaces both in cosmetic medicine and in traumatology and dentistry.

Nanoceria also has an antitumor effect [280]. This property is most studied in relation to MCF-7 breast cancer cells, the dose-dependent cytotoxic effect for which it was selective [281,282,283]. A similar result was obtained when studying the effect on HT-29 colorectal cancer cells. It is noteworthy that an increase in cytotoxicity was observed when a hollow nanoparticle coated with SiO_2_ was formed [284]. Another option for realizing the properties is the use of cerium dioxide derivatives as a means of photodynamic therapy, which was confirmed in the experiment with irradiation of triple negative breast cancer MDA-MB-231 and melanoma A375 cells by radiation with a wavelength of 465 nm [285]. Also, nanoceria can increase the sensitivity of L3.6pl pancreatic cancer cells to radiation exposure, which may be due to differences in local pH value [286]. In addition, cerium oxide nanoparticles can be loaded with an antitumor drug and thus play the role of a drug delivery system [287].

The spectrum of antibacterial effect of cerium dioxide nanoparticles extends to both Gram-positive and Gram-negative bacteria. In particular, the literature describes the effectiveness against such microorganisms as *Escherichia coli* [273,288,289,290], *Bacillus subtilis* [290], *Staphylococcus aureus* [288,289], *Pseudomonas aeruginosa* [289,291,292], *Salmonella typhimurium*, *Listeria monocytogenes*, *Bacillus cereus* [288], *Porphyromonas gingivalis*, and *Prevotella intermedia* [293].

Based on this, we can conclude that cerium dioxide nanoparticles have a wide range of actions and, although not yet used in biomedicine, are promising for further development.

## 4. Possible Directions of Modification of Rare Earth Metal Nanoparticles

Rare earth metal nanoparticles can be modified in several ways: conversion to a different oxidation degree, addition of excipients, creation of nanocomposites (discussed in the sections on the corresponding metals), formation of bioconjugates, and modification of particle morphology.

The first method is applicable for metals having variable oxidation degrees. At the same time, their nanoparticles, with the exception of cerium, can be stable in an uncharacteristic degree of oxidation mainly under specific conditions, which makes it impossible to use them in biomedicine [294]. 

Several ways of modifying lanthanides with excipients have been described in the literature. For example, the addition of excipients such as glycols allows the morphology, solubility, density, and optical properties of nanoparticles to be tuned [295].

For gadolinium, which is insoluble in oxide and toxic in ionic form [18], PEGylation using a microwave method has been proposed, leading to its solubilization [296]. In the course of further experiments using gadolinium, nanoparticles incorporated into liposomes with a targeting effect on pathologically altered vascular tissue were produced [297]. It is worth noting that dendrimers are considered to be an equally promising option for gadolinium modification. It is likely that their size (5–10 nm) contributes to better accumulation of substances in the tumor microenvironment. These technological methods can be used both in targeted drug delivery systems and for diagnostic purposes [298,299].

More complex pharmaceutical compositions have been investigated using ytterbium, gadolinium, and europium as examples. Hybrid micelles chemically linked to a complex of lanthanides and cyclenes were prepared. In evaluating the efficiency of accumulation of these compounds in tumor cells, experiments were performed with cultures of HeLa-type cervical cancer cells and mice with HeLa cell xenografts. Positive results were achieved for gadolinium and ytterbium [300]. For all the promising effects of gadolinium, a risk of systemic inflammatory reaction and fibrosis has been identified. The presumed cause of this phenomenon is the degradation of complexes under in vivo conditions. As a consequence, there is a need not only to use carriers that provide targeted action but also form strong chelates with rare earth metals [301]. An example of such a high-strength compound is an organometallic ytterbium nanoparticle bound to ferrocin and mannose. Ytterbium retained antitumor activity, while mannose provided release only during binding to the corresponding specific receptors of hepatocellular carcinoma cell line HepG2. The authors of the study emphasize that the therapeutic efficacy of this combination has been established only in vitro and requires additional study [302]. It is also inappropriate to project the results obtained on any combination of carbohydrates with lanthanides. In a study of the degree of binding of chitosan-modified D-mannose, it was observed that the strength of the complex varies significantly for metals with the same degree of oxidation. In particular, ionized europium is inferior to other trivalent metals, which requires further comparison of stability with micro- and ultramicroelements of the human body before in vivo application [303]. A similar situation is observed in attempts to combine lanthanides with alginate [304]. Anionic derivatives of cyclodextrin, on the contrary, have high biocompatibility in combination with the ability to reliably chelate lanthanides. However, this modification has another limitation: it has only been studied for MRI imaging applications, so we can neither confirm nor deny the retention of any pharmacological effects [305]. The main results of studies of interaction between rare earth metal nanoparticles and excipients are summarized in Table 1.

As a possible way to increase the biocompatibility of rare earth metal nanoparticles, it should be proposed to create their pharmaceutical compositions with biopolymers. These include such polymers as dextran [273], chitosan [306], alginate [307], hyaluronic acid [308], carboxymethylcellulose [309], collagen [310], and gelatin [311]. According to the results of studies of compositions of nanoceria and biopolymers with human fibroblast cell cultures, no signs of cytotoxicity were revealed [273,306]. At the same time, not only the preservation of antioxidant activity, but also a pronounced regenerative effect was revealed. Notably, metabolic activation prevailed over proliferative processes [273]. At the same time, a study by Verdiana Marchianò et al. described the need to increase the nanoparticle content when developing complex formulations containing PLGA-encapsulated nanoceria in gelatin due to the presumed decrease in particle availability [311].

Thus, we can conclude that the strategy for selecting excipients in the research of rare earth metal nanoparticles as agents for biomedical applications includes the following main points: (1) Ensuring nanoscale. For this purpose, it is advisable to use substances with stabilizing properties (e.g., dextran stabilizing nanoceria) [273]. Depending on the particle size, both high biocompatibility (75 nm) and non-selective cytotoxic effect (100–160 nm) can be observed [312]. (2) If the risk of adverse reactions is high, preference should be given to excipients that form strong complexes with nanoparticles and thus slow down the release of the active substance and, consequently, the risk of acute toxicity [307]. Alternatively, we can consider the development of targeting systems that ensure the accumulation of nanoparticles in pathologically altered tissues [297,302]. (3) Preference should be given to biocompatible and biodegradable excipients that give the surface a positive charge, and excessive excipients should be avoided so that the pharmacological effect is not reduced [274,313].

Rare earth metal nanoparticles can be modified by bioconjugation. Currently, bioconjugation using interleukins, antibodies, and RNA has been described. Bioconjugation with interleukins 17, 13, and 8 is mentioned in the literature. A gelatin hydrogel coated with interleukin 17 demonstrated anti-inflammatory activity in an animal model of encephalitis [314]. Coating of gadolinium nanoparticles with interleukin 13 peptide increased the capture of the particle by tumor cells in a mouse model of glioblastoma [315]. It is possible to develop more complex compositions including nanoparticles containing ytterbium, erbium, gadolinium, and yttrium electrostatically bound to particles loaded with docetaxel, interleukin 8, and small interfering RNA. This multimodal platform allows simultaneous tumor imaging, gene therapy, and chemotherapy [316]. Conjugation with RNA has also been described for nanoceria. Conjugated with miR-146a, nanoparticles enhanced diabetic wound regeneration according to Carlos Zgheib et al. [317].

Bioconjugation with antibodies of rare earth metal nanoparticles has been described for europium, lanthanum, and cerium. All of them were conjugated with antibodies to interleukin 6. This immunoassay method has been characterized by researchers as fast, reproducible, highly sensitive, specific, and suitable for serum assays [318,319,320].

An alternative direction of modification is to change the morphology of nanoparticles. In addition to nanoparticles, nano-objects also include nanorods [321], nanocubes [322], and nanotubes [323]. It is noteworthy that the production of nanorods and nanocubes follows an identical technique and can be controlled by microwave radiation time. However, the morphology of nanorods should be prioritized due to their higher redox activity [322].

Cerium-aspartic acid nanotubes have also been described in the literature. It was observed that it was the combination of asparagic acid addition and tube morphology that allowed the catalytic activity of the colorimetric reaction to be achieved in the determination of cysteine levels, which was not found for the Ce (NO_3_)_3_ precursor. Thus, this nano-object can be used as a highly sensitive nano sensor for the determination of cysteine content [323], which has diagnostic value in chronic kidney disease [324]. In addition to new applications, changes in morphology may enhance the already known activity of the rare earth metal. Michelle de P. et el. described an enhanced antibacterial effect of cerium-gallium titanate nanotubes against *S. aureus* and *E. coli* microorganisms. According to the authors of the study, this was due to the increase in specific surface area relative to the nanoparticles [325]. Cerium-silver nanorods are also currently known. According to the authors’ conclusions, this approach contributes to the long-term preservation of photocatalytic activity of the object [326].

Hybrid gadolinium oxide nanorods were also developed, which have a pronounced antibacterial effect against *Escherichia coli*, *Klebsiella pneumonia*, *Staphylococcus aureus*, and a small effect against *Micrococcus luteus*. It should be noted that no comparison has been made with the nanoparticle form, so a comparison of efficacy is not possible [327]. Europium nanorods demonstrated an improved safety profile in in vivo experiments. There was no significant accumulation of europium in liver, spleen, kidney, and lung tissues [328], which we have previously mentioned for europium nanoparticles.

It should be noted that other morphological variants of nano-objects have also been developed for other representatives of lanthanides, for example, nanoneedles and nanopolyhedrons of lanthanum oxide, but they have not been considered in the context of biomedical applications [329].

Thus, we can conclude that currently the most promising direction of modification we can consider is the use of excipients, as they allow us to reduce the risk of toxicity, increase the stability and biocompatibility of particles, and ensure targeting. At the same time, the main disadvantage of this direction is the difficulty in selecting the excipient, as there are no unambiguous methodological recommendations on this issue at the moment. Modification by bioconjugation is likely to enhance the intrinsic pharmacological effects of nanoparticles, increase targeting, and in some cases, expand the scope of application. At the same time, the published information in this area is still very limited in terms of the range of possible applications. Another significant disadvantage is undoubtedly the great complexity of working with immunobiological substances in relation to excipients. The main advantage of morphology modification is the large specific surface area, which theoretically favors the increase in particle activity. However, this approach does not exclude the use of excipients and also requires additional comparative studies. A similar problem is observed in the development of nanocomposites, where an additional difficulty is the difficulty of assessing the contribution of each component to the therapeutic and unwanted effects.

Thus, we can assume that possible future directions for the development of drugs containing rare earth metal nanoparticles could be hybrid micelles, as well as modified formulations of oligomers and polymers.

## 5. Discussion

This work does not comparatively analyze the efficacy of rare earth metal nanoparticles and drugs, nor does it consider nanoparticles in the context of their application limited solely to the delivery of other drugs. After analyzing all the above results of published studies, we can come to several conclusions:

(1) The main applications of rare earth metal nanoparticles are MRI diagnostics [111,112] and therapy of malignant [59,230,286] and infectious diseases [68,69,273,288].

(2) Safety of rare earth metals applications in medicine. At present, the information about possible risks of application is contradictory [178,186,215]. There are references to both protective [177] and regenerative properties [72,95,206,292] and cytotoxicity [72,189]. Less frequently, we can find information about selective cytotoxicity, particularly against tumor cells [77,78]. At the same time, nanoparticles of rare earth metal oxides are safer than their ionic forms [18].

(3) Possible solutions to the problem by assessing risks and benefits. Based on the first point, we can identify the preferred directions for the development of metal nanoparticles for medical applications. Thus, rare earth metal nanoparticles can be used for external application as antibacterial agents [68,155,241,242], which significantly reduces the risks of systemic unwanted reactions. Another possible direction is the use of some lanthanides for MRI imaging, which is usually not performed on a regular basis, thus reducing the risk of at least manifestations of chronic toxicity [43,66]. Finally, it is reasonable to investigate the possibility of use as an antitumor agent, provided that clinical efficacy is consistent with results obtained on cell cultures and in vivo, or if targeting is possible [147,196].

(4) Improving the safety profile through changes in technology. The rational choice of excipients or their combinations, as well as the combination of nanoparticles with other modern dosage forms and targeted delivery systems, allows for an increase in the targeting of the target (e.g., tumor) [298,299,302]. This reduces the risk of dissociation and thus cumulation of metals in tissues [305] and reduces the dosage due to the increased accumulation of nanoparticles at the point of application of the effect [300]. The size of the nanoparticles themselves also plays an important role since their cytotoxicity depends on it.

Certainly, for many rare earth metal nanoparticles, additional independent in vitro and in vivo studies are required before it is possible to draw unequivocal conclusions about the prospects for their application in medicine and pharmacy. At the same time, for metals already introduced into clinical practice (such as gadolinium and yttrium), as well as the most studied by modern science (for example, cerium), the format of oxide nanoparticles may allow for an expansion in the range of therapeutic action, as well as to increase the safety and efficacy of therapy.

## 6. Conclusions

In accordance with all of the above, we can conclude that the most studied rare earth metals as agents for medical use are nanoparticles of gadolinium, yttrium, cerium, ytterbium, and lanthanum oxides. However, the question of the balance between the benefits of their use and the risks accompanying it is still acute. Despite this, the application of rare earth metal nanoparticles is quite explored in such areas as visualization and treatment of malignant neoplasms, as well as therapy of bacterial infectious processes. In this regard, further work to confirm these effects in clinical trials and the selection of safe doses is reasonable. 

## Figures and Tables

**Table 1:** The result of the interaction of excipients and rare earth metal nanoparticles.

Metal	Excipient	Class of Compound	Effect	Type of Study	Sources
Gadolinium	Polyethyleneglycol	Biopolymer	Solubilization	In vitro	[296]
Gadolinium	Liposomes	Fatty substance	Targeted action on pathologically altered tissue	bEnd.5, RAW	[297]
Gadolinium	Polyamidoamine dendrimer	Biopolymer	Increased accumulation in the tumor microenvironment	In vivo	[298,299]
Gadolinium	Cyclen	Hybrid micelles	Increased accumulation in tumor cells	HeLa, in vivo	[300]
Ytterbium	Cyclen	Hybrid micelles	Increased accumulation in tumor cells	HeLa, in vivo	[300]
Ytterbium	Ferrocene and mannose	Metallocene and monosaccharide	Targeted antitumor	In vitro HepG2	[302]
Ytterbium	Anionic cyclodextrins	Oligomer	High strength of the complex	In vitro	[305]
Europium	Cyclen	Hybrid micelles	No effect	HeLa, in vivo	[300]
Europium	Chitosan and mannose	Biopolymer and monosaccharide	Low strength of the complex	In vitro	[303]

## Data Availability

Data sharing is not applicable.
